# Senescence: The Compromised Time of Death That Plants May Call on Themselves

**DOI:** 10.3390/genes12020143

**Published:** 2021-01-22

**Authors:** Matin Miryeganeh

**Affiliations:** Plant Epigenetics Unit, Okinawa Institute of Science and Technology Graduate University, 1919-1 Tancha, Onna-son, Okinawa 904-0412, Japan; matin.miryeganeh@oist.jp; Tel.: +81-804-207-4224

**Keywords:** senescence timing, aging, climate change, reproductive synchrony, productivity

## Abstract

Plants synchronize their life history events with proper seasonal conditions, and as the fitness consequences of each life stage depend on previous and/or subsequent one, changes in environmental cues create cascading effects throughout their whole life cycle. For monocarpic plants, proper senescence timing is very important as the final production of plants depends on it. Citing available literatures, this review discusses how plants not only may delay senescence until after they reproduce successfully, but they may also bring senescence time forward, in order to reproduce in favored conditions. It demonstrates that even though senescence is part of aging, it does not necessarily mean plants have to reach a certain age to senesce. Experiments using different aged plants have suggested that in interest of their final outcome and fitness, plants carefully weigh out environmental cues and transit to next developmental phase at proper time, even if that means transiting to terminal senescence phase earlier and shortening their lifespan. How much plants have control over senescence timing and how they balance internal and external signals for that is not well understood. Future studies are needed to identify processes that trigger senescence timing in response to environment and investigate genetic/epigenetic mechanisms behind it.

## 1. Senescence and Aging in Plants

Senescence (from the Latin word “senēscere”: to grow weak, become exhausted, and to be in a decline) generally refers to the process of growing old and is associated with decay and mortality or decreased fertility with age [1], but it is actually a very widespread concept for plants. Plants have specific characteristics that violate the general, classical definition of senescence. For example, they are modular; meaning their architecture is made of a repetition of units which allows them to rejuvenate [2]. In addition, their cellular division does not always cause shorter telomeres [1]. There are even some plants for which the concept of senescence simply does not apply. Researchers addressed the hypothesis of senescence that assumes aging results from an accumulation of deleterious mutations, by studying extraordinary long living trees: bristlecone pine (*Pinus longaeva*), ranging in age from 23 to 4713 years. They studied viability traits, such as seed weight and germination rate, biomass, and frequency of mutations and found no significant relationship between these factors and age of the trees. They concluded that these trees do not senesce [3]. Another group of researchers [4] examined whether an extraordinarily long-living herb *Borderea pyrenaica* (Dioscoreaceae), which is known to live more than 300 years, experiences senescence. They investigated the relationship between age, reproductive value, and vital rates. No evidence for senescence was found as growth and fecundity did not decrease at older ages, and survival and reproductive value increased with age. Another study on these perennial herbs [5], tested age-related changes in several photo-oxidative stress markers and found no age-dependent signs of oxidative stress. Therefore, they suggested that age-induced senescence is not a universal feature of aging in perennial plants.

It is true that as organisms grow old, their performance declines. This might raise the question of why natural selection is not replacing individuals that perform poorly at older age by the ones performing stronger as aged group? The fitness decline caused by senescence has been suggested to be either because of accumulation of mutations [6] or results from genes that have been chosen by natural selection because of their positive effect in earlier life, despite of their adverse effects in later life (pleiotropy) [7]. The classic theory of senescence evolution says that the power of natural selection decreases with age [6]. Hamilton W.D. (1966) [8] discussed that increased mortality and/or decreased fertility in older ages does not affect the fitness as much as it would have, had those happened at younger ages. He suggested that, as the individual grows old, mutations that have caused better performance and increased fertility at younger age, but have caused less performance later on in life, will be established in population, because high performance at early life is of a great advantage for plant and it is selectively favored [7,8,9]. In other words, mutations coding for “live fast, die young” performance, are favored and naturally selected over genes coding for average performance and longer life [10]. Senescence, in the other hand, is considered to have been evolved as an essential strategy associated with plant reproduction, adaptation, fitness, and survival [11]. Various gene expression profiling and transcriptome studies have shown a conserved pattern among plant species for senescence regulation, as similar catabolic pathways were found to be upregulated at senescence. This shows that senescence could be an evolutionary selected trait [12,13]. We know that plants not only delay death until after they reproduce successfully [14,15], but they sometimes even bring senescence time forward, in order to escape upcoming unfavored environmental conditions and maximize their productivity [16]. In fact, plants have been called “unusual organisms” that can have some control over their own life span based on environmental cues, above and beyond the aging process [14,15,17].

## 2. Age-Dependent/-Independent Senescence in Plants

Even though senescence is part of the aging process [18], it does not necessarily mean plants always have to reach certain age to senesce. Recent studies have proposed that the timing of whole plant senescence is influenced by developmental age more than calendar age [16,19]. Besides age-dependent/developmental senescence, environmental conditions can also trigger senescence, and it has been shown that the timing and rate of senescence is highly affected by environmental cues such as photoperiod, temperature, and moisture in soil [18,20,21]. Therefore, studying senescence in natural populations is complicated because it is influenced by environmental factors that fluctuate seasonally or even daily and may also influence age-dependent mortality pattern [22]. To study the age-dependent/-independent dynamics of senescence, a group of researchers [23] investigated demographic aging in natural populations of *Plantago lanceolata* (Plantaginaceae), and reported synchronous changes in senescence across four cohorts (a cohort is a group of individual plants of same age) over time (i.e., environmental dependent senescence). Another study [24] of size-based/age-based senescence on *P. lanceolata* confirmed the Hamilton prediction that says the impact of selection decreases with age. Their analyses showed decline in size, lower inflorescence production, and reduced physiological strength prior to death, which were all best explained by size rather than age suggesting an important role for the environment in determining senescence.

Other researchers have shown that both initiation and termination of flowering (final senescence) are sensitive to environmental conditions [16,17,25,26], which give plants the advantage of flexibility in response to changing environment and allow setting seeds and senescing at a suitable time. In two recent publications, using groups of *Arabidopsis thaliana* that differed in age, it was also shown that whole senescence in plants is strongly synchronized with their environmental condition All groups set seeds and senesced at the same time regardless of their age [16,17].

Studies using natural population of *A. thaliana* have identified genes that are involved in both local adaptation and senescence, and suggested that senescence may be helping with adaptation [27]. Under stressful environmental conditions (if the developmental time is appropriate), nutrients from vegetative organs such as leaves, reallocate towards reproductive organs. This is an important adaptation trait that plants have evolved, in order to accomplish their life cycle even under undesirable conditions [28]. Whether plants enter the senescence phase to avoid the stressful situation or they compromise for shorter life in exchange of better final outcome (as yield and seed set) is yet to be discovered. Compare to phenological studies focusing on bolting and flowering, whole-plant senescence and the effects of environmental changes on its patterns are still not well understood, because the timing of this last developmental stage is influenced by multiple factors affecting all previous developmental stages which makes it complicated [13,29].

## 3. Whole Plant Senescence

There are two main types of senescence in plants. The sequential or organ senescence, which happens in a continuum pattern at a certain developmental stage after accomplishing certain tasks, when senescing organs recycle their extra nutrients towards developing and growing ones [30]. For example, spring flowering is the result of consuming relocated nutrients from senescing autumn leaves. Organ senescence is mostly associated with age, but also with environmental condition [31]. The second type of senescence is reproductive senescence, which leads to the whole plant senescence in monocarpic plants and is usually called “monocarpic senescence”. It is the final stage of development and helps with final production and seed quality [32], and the way it is precisely programmed to occur after distinct sequential developmental phases, is very unique to monocarpic plants. Reproductive senescence initiates a gradual death and has been called the “natural cause of death” in plants [33]. Although most senescence studies have focused on leaf senescence rather than whole plant senescence, but in monocarpic plants leaf senescence is actually coordinated with whole plant developmental phases including the whole plant senescence [21,34].

Timing of whole plant senescence in monocarpic plants is important for fitness and natural selection, as close to the end of reproductive phase, plants try to invest all their resources and nutrients in final production [35]. One of the earliest observations of monocarpic senescence in plants was by Hildebrand (1881) [36], when he suggested that whole plant senescence happens after plants accomplish the reproductive phase, which is itself the result of remobilization of nutrients from vegetative to reproductive organs in order to provide resources for developing seeds. Therefore, in case of monocarpic plants, flowering senescence (“floral arrest”) is followed by whole senescence and can be studied as a senescence factor. An obvious definition for whole flowering senescence would be the time point when all the flowers are senesced and no more “flowering initiation” will occur [16,17,26]. Flowering senescence has an important role in determining the length of reproductive period, and also it affects the reproductive potential such as optimization of fruit and seed production. One of the first studies focusing on “flowering termination” and whole senescence was done over 26 years ago, when Hensel (1994) [37] studied the relationship between the proliferative capacities of inflorescence meristems and final fruit development in *A. thaliana*, and provided strong evidence that floral arrest is mediated by a communication between inflorescence meristems and developing final fruits and seeds. A recent study of floral termination in *A. thaliana*, expanded the classic model of Hensel, and examined the mechanism by which final fruits affect flowering termination [38]. They suggested that inflorescences only arrest at certain developmental age and in response to a highly localized auxin signal from recently produced fruits. Another group of researchers [39] investigated the correlative control of *A. thaliana*’s seeds over inflorescences and studied how reaching to a certain number of seeds inhibit further maternal growth. They identified expression of stress- and senescence-related genes right after fruiting and inflorescence meristem arrest. They also reported sudden arrest in mitotic activity upon fruit removal, meaning the term “mitotic senescence”, a proposed name for growth arrest after fruit production when meristem cells lose their ability for mitotic cell division [31,40,41], may have not been used properly. In *A. thaliana*, it is shown that producing certain number of flowers and fruits will lead to reproductive meristem arrest and if fruit numbers are low, inflorescence meristem continues its activity and can also be reactivated in case of fruit removal [38,42].

A correlation control between developing fruits/seeds and senescence timing in monocarpic plants has been also observed when removal of reproductive structures or preventing their development, delayed terminal senescence [40,43,44,45,46]. This is suggested to be either through source–sink relationship [43] or in another point of view, via signals from offspring [44,47,48]. However, recent studies have shown that the connection is more complicated than that [49]. Several quantitative trait loci (QTL) analyses using recombinant inbred lines (RILs) populations of *A. thaliana* have reported accession- and/or condition-specific QTLs for advancing or delaying senescence (see, e.g., in [50,51]), suggesting plants have evolved natural genetic variations according to their evolutionary and also ecological history. Woolhouse [52] suggested that because of polyphyletic origin of monocarpic senescence, there may have been independently evolved control strategies in different plant groups and encouraged scientists to avoid generalizing and simplifying the concept of monocarpic senescence and instead explore senescence separately in different species. Investigating senescence in pea plants (*Pisum sativum* L.) led scientists to reject the simplified source–sink view that says senescence is induced by developed flowers and fruits [30]. They suggested that senescence is the “consequence” of reproductive phase; meaning the commitment of plant to redirect the nutrients towards reproductive sinks is “required” but “not enough”, and monocarpic senescence timing is influenced by many factors including environmental condition and also the previous developmental life stages. For example, low nitrogen level has been reported to induce early senescence [53], as opposed to high nitrogen level which delays senescence [54]. Studying the effect of day length on senescence, as one of the main environmental factors, using different ecotypes of *A. thaliana* showed that long day only causes earlier senescence in early flowering accessions, and not in late flowering accessions, which shows senescence being influenced by both environment and genetic [55]. They also found that senescence was linked to other developmental traits such as flowering and fruit number, which was evidenced in other studies as well where correlation between flowering time and seed set/senescence was reported (see, e.g., in [56,57,58,59,60]). They suggested that the effect of genetic and environment on senescence and related developmental traits might be through common regulatory pathway as the pattern of association between senescence and other traits was the same, regardless of senescence variation being caused by ecotype or day-length [55]. This was seen in similar cases before, where QTL studies using *A. thaliana* RILs reported overlapping flowering and senescence genes with the loci affecting either of those traits and suggested senescence and flowering may be genetically linked and sharing regulatory loci [17,61]. Other lines of studies have shown that senescence of first few emerging leaves, will send nutrients such as nitrogen to later-emerging leaves and affect whole plant senescence [50,62,63]. Studying 45 accessions of *A. thaliana* and 155 RILs also showed that *A. thaliana* plants may have evolved to use various methods to accomplish developing fruits, seeds, and then senesce, which seemed to be dependent on flowering time [51]. Later-flowering groups used reallocated nutrients from senescing leaves, whereas earlier-flowering group of plants used photosynthates. However, even when senescence is flowering-dependent, it does not necessarily follow the pattern in which later flowering means later senescence, yet it might mean plants adjust their flowering time, in order to coordinate their senescence time with appropriate environmental condition that is in interest of plant fitness and productivity [16,17,26]. Even though experimental studies have shown that removal of reproductive organs will prolong vegetative phase and delay flowering, which then may increase plant life span and delay senescence, this may not simply be the case. In order to do the proper adjustment with the environment, flowering and senescence seem to interact with each other, and studies have shown that whole plant senescence is associated with both flowering-dependent and flowering-independent pathways [16,17,29,34,42,61].

## 4. Plants Schedule Their Life Events Based on Environmental Signals

In order to reach the optimal phenotypic state and therefore eventually optimum productivity, plants have evolved the ability to sense seasonal cues and alter their developmental responses accordingly. This process is called seasonal developmental plasticity [64]. Because of their sessile nature, plasticity is probably the most efficient way for plants to change their environment. Even though they cannot move and change their habitat, they do change their exposure to it through phenotypic response to environmental cues [65]. They carefully time their life history events to overlap and synchronize with favorable environmental conditions in order to increase reproductive success and maximize fitness [66,67,68]. Thus, plants need to make important developmental decisions, such as when to germinate, when to shift from vegetative to reproductive phase, when to fruit, and finally when to senesce. They enter the reproductive phase by flowering (floral transition), and schedule to exit the reproductive phase at the proper time as well (floral termination or senescence) [16,26]. This means environmental changes will influence the expression of their developmental traits which in turn may cause strong natural selection on those traits and evolutionary responses that depend on genetic and/or epigenetic variation that may even be inherited by next generation [69,70].

Two of the most important environmental factors that affect the transition of plants from one life stage to another are temperature and day length, and they are usually considered together as photothermal value. Some species only flower if they receive a certain threshold of photothermal units (PTU), and in some even the duration of flowering is affected by PTU, that then consequently affect the timing for next key life stages such as senescence [16,57]. Forecasted climate change is expected to shift seasonal condition for plants, and therefore shift the timing of life history events [71,72,73]. For example, plants that require vernalization for flowering will have to adapt and change behavior, as warmer winter will reduce exposure to vernalization and shortens growing seasons, while increased summer drought is likely to reduce survival through subsequent seasons [74]. Studies are already showing adaptive responses of plants to climate change and alteration in timing of their life events. For example, earlier flowering in *Linanthus androsaceus* (Polemoniaceae) has been reported in response to early drought season [57]. Some species are advancing their phenology time, with earlier bud bursting and flowering [75,76,77,78,79]. Some researchers have also reported loss of vernalization requirement and shortening lifespan in response to environmental changes when earlier reproduction and earlier senescence is favored [80,81,82]. Environmental temperature during seed set has also shown to influence life history via modulating seed dormancy, and therefore affecting germination timing [83,84,85].

Most studies about environmental influence on plants, have focused on germination and flowering time [71,86] and less is known about the effects of seasonal changes on flowering termination time and whole plant senescence. Even though focusing on one event has the advantage of evaluating the selection effect directly on that event, but the reality is that in nature, plants are treated as a whole organism. Their life stages influence one another and are inherently linked meaning environmental changes can create cascading effects throughout the life cycle, and often the fitness consequences of each transition depend on previous and/or subsequent one [65,74,86,87]. Therefore, germination timing, flowering, and senescence time are all connected to each other and need to be studied as a whole story of plant’s life in order to be understood and interpreted correctly.

The first life history event which responds directly and sensitively to environment is germination timing and it has key cascading effects on the rest of developmental phases throughout life cycle [88,89,90,91]. It affects the success of establishment for young seedling and also has impact on growing and developing of plants, as it determines the balance between how much time there is to collect resources for reproduction and how favorable the seasonal condition will be throughout life span [74,92,93]. Specially, when chilling period over winter is required for flowering, which is the case for many annual plants, proper germination timing will arrange the exposure to sufficient cold for flowering that can affect the schedule for senescence and setting seeds later on. Studies have reported changes in germination timing with different climates [94,95]. In addition, there is usually variation in germination timing among individuals of natural annual plant populations [79]. Therefore, experiments that manipulate germination timing can help us investigate its impact on subsequent developmental phases including senescence in face of upcoming climate change.

Donohue (2002) [65] conducted an experiment where germination timing in five natural populations of *A. thaliana* was manually planned (i.e., seeds were forced to germinate at different times of the year: in early autumn, later autumn, and early spring), and significant changes in post-germination traits including reproduction time were found, which therefore led to changes in fitness. Interestingly, later autumn germinant compensated delayed germination by slightly faster vegetative growth than early autumn germinant, therefore least size differences among cohorts was observed. Spring germinant failed to reproduce at all, indicating the importance of choosing right time for germination according to the seasonal environment. That experiment confirmed that germination timing can influence the selective environment experienced at later life stages as it has also been suggested before [96,97]. Another study using RILs of *A. thaliana* investigated plasticity of life history traits in response to germination timing variation and possible changes of natural selection on them. Strong alteration in timing of life history traits according to time of germination was found, and novel adaptive genotypes created by seasonal changes of germination timing were reported [98]. Another group also investigated the effects of germination timing on upcoming traits using two populations of *Streptanthus tortuosus* (Brassicaceae) which show a high variation in timing of germination and reproduction. They also concluded that shifts in germination timing influences the expression of subsequent traits including senescence and also affects fitness of the plant [74].

The next major life history phase after germination is flowering. Flowering time determines the environment that plants will be experiencing at the time of senescence and is itself affected by germination time. Wilczek (2009) [86] showed that depends on germination timing, *A. thaliana* accessions switch their flowering time to either before or after winter and developed a photothermal model that predicts flowering time. Another study [79] extended Wilczek’s photothermal model and included the reproductive phase and predicted seed set. They used a natural population of *A. thaliana* that were germinated and grown at different times of the year and reported that the timing for setting seed and whole plant senescence depend on environmental temperature and if the temperature increases, plants both flower and set seed earlier. They suggested that temperature control of flowering time is a way that plants also control the timing for setting seeds and senescence [79].

## 5. The Art of Senescence Synchrony and Harmonizing with Environmental Cues

The level of synchronization of reproductive timing relative to germination timing and the concept of “senescence synchrony” were first introduced using another study that manipulated germination timing [16]. They performed a sequential seeding experiment (SSE) in which seven cohorts of *A. thaliana* which were each one week older than the next one were compared to each other for the degree of reproductive synchronization. The difference in germination timing caused desynchronization of flowering in early flowering accessions and yet they all showed senescence synchronization (i.e., they set seeds and senesced all at the same time). Therefore, they concluded that timing of flowering termination and whole-plant senescence is regulated internally in response to seasonal environment and it is independent of age and flowering initiation time. In addition, upregulation of senescence-related genes at the synchronized senescence time for all cohorts was observed. Even though they used least senesced leaves for gene expression analyses, upregulation of senescence-related genes was seen even 2–4 weeks before whole senescence, which may be a sign of plants reallocating resources to reproductive organs. They suggested that under uniform environmental conditions, plants of different ages can synchronize senescence according to their environment, even if they differ in age.

**Figure 1:**
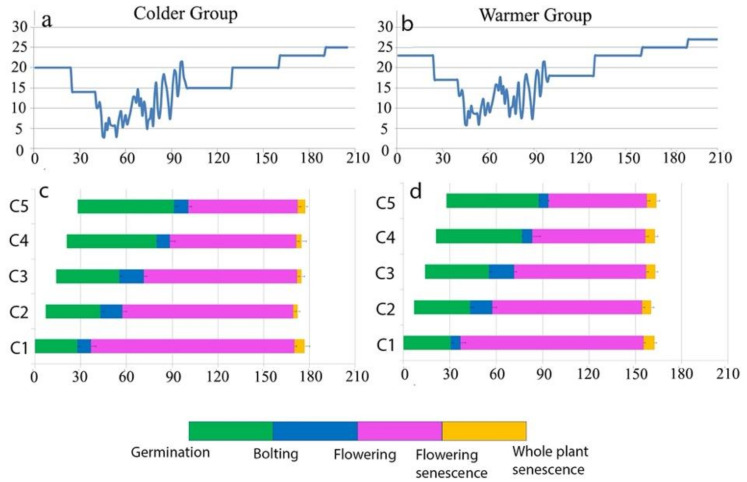
Results of Sequential Seeding Experiment (SSE) for Cvi accession of *A. thaliana*. Phenological responses of the five cohorts (C1–C5) in Colder Group (**c**) and Warmer Group (**d**) are indicated by bars representing the periods between germination and the four successive reproductive timings. Colors correspond to those in the bar at the bottom of the figure. The *x*-axis shows the calendar date (number of days since germination). The *y*-axis in the top panels show the temperature for Colder Group (**a**) and Warmer Group (**b**), and the y axis in the bottom panels refer to cohort number 1–5 (C1–C5). This figure is visualizing 5 cohorts of *A. thaliana* (Cvi) which are each one week older than the next one that were divided to grow in two different temperature regimes. Colder group (left) lived about a month longer than warmer group and yet the cohorts within each group synchronized senescence timing with each other and not with their same age replicates in the other group. This emphasizes the role of environmental condition on senescence timing (Adopted from Miryeganeh 2020 [17]).

In a follow up study [17], they reproduced two sets of that sequential seeding experiment in controlled environments, using two greenhouses with two different temperature regimes (colder and warmer; Figure 1) and monitored whether the cohorts will flower and senesce based on their age or their environmental conditions. While flowering was desynchronized among cohorts, striking synchrony in senescence timing among cohorts within each greenhouse and not with their counterpart replicates of same age in the other greenhouse was found. Any age replicates synchronized senescence with older and younger ones within each green house, by adjusting their flowering duration. In the “colder” group, where plants experienced lower temperature, the flowering period lasted longer, and flowering senescence occurred later compared to “warmer” group. As all cohorts within each greenhouse synchronized senescence with each other and not with their replicates growing in the other greenhouses, this supported the hypothesis that seasonal conditions have a greater impact on senescence timing than age, and it shows that plants control senescence by adjusting the timing of life-history events based on the environment in which they are growing (Figure 1).

They then conducted a QTL analysis using *A. thaliana* RILs to look for genetic regions potentially associated with senescence synchrony, which one may argue was a counterproductive attempt as senescence synchrony, resulting in low variation in senescence timing among lines that then led to weak QTL detection for senescence. This suggested that small-effect QTL is in favor of synchronized behavior to secure senescence synchrony under desirable environmental condition [17]. In both of these studies [16,17], plants were able to synchronize senescence via changing the length of their developmental phases especially length of flowering period (reproductive phase). Shortening flowering period in cohorts that germinated later in the season allows the reproduction before hot and dry season; similar strategy that is also seen in crops when they complete their life cycle before the hot season. Mediterranean crops such as barley and wheat complete their developmental life cycles and senesce, before summer when they experience longer days as a seasonal cue [99].

Therefore, the life-history theory that assigns a fixed schedule for reproductive phases is not always true, and plants adjust their lifespan in response to their environment. They manage their senescence timing according to the accomplishment of their reproduction, even if that means to shorten their lifespan, set seeds, and senesce not long after flowering [14,16,17,100]. Skulachev (2001) [101] referred to senescence and programmed cell death as the “Samurai Law of Biology” (“it is better to die than to be wrong”) which is a view of senescence being an unavoidable developmental phase that allows plants to program their self-deterioration and control their own “time of death” when they sense dying at the right time/condition overweigh living at the wrong time/condition [102]. In addition, even though plants try to shift their life history events and match them with proper environmental condition, the shifting of schedules may also change the adaptive value of those traits. If plants choose particular conditions to germinate, flower, set seed and senesce, it will affect the selective environment that determines the evolution of those key developmental phases and therefore genetically based associations can evolve for those life history traits [65,89,96]. Thus, proper decision about senescence timing is an essential evolutionary characteristic that affect productivity, adaptation and evolution of plants.

## 6. Senescence Timing and Productivity in Plants

When monocarpic plants enter the reproductive phase and all their organs gradually die, the nutrients translocate either to the developing seeds that can germinate and initiate the next life cycle and/or towards storage organs [103,104,105,106,107]. A simple assumption in almost all studies that have focused on the relationship between plant productivity and senescence is that delayed senescence translates to more photosynthetic life and therefore more productivity [108]. Whole-plant senescence has shown to have an important role in crop productivity [107], and grain number and weight in crops also have shown association with the onset of senescence [109]. In cereal crops, there is coordination between leaf senescence and starting of seed maturation [110,111]. In addition, late-senescing or stay green varieties often show higher yield [106,112,113].

However, it should be noted that even though delayed senescence may increase the productivity because of longer photosynthetic period, the timing, and the length of senescence process which affect the duration of reproductive period, still needs to be well coordinated and balanced with previous developmental phases especially with flowering time [29,106,114,115]. In addition, senescence timing is under strong influence of environmental cues and increasing productivity depends on many different factors which plants are naturally managing as part of their effort for adaptation. For example, delayed senescence mutants of wheat results in higher amount of Nitrogen in the grain, although it has no effect on total yields [116], which shows even though delayed senescence may mean more nutrients assimilation, it is not always an improvement in productivity [15]. In barley and wheat, lines with functional senescence genes showed earlier senescence and more grain protein, and yet reduced yields. A delayed senescence has also shown to be negatively correlated with protein concentration in cereals, despite of higher yield/grain weight [117,118,119,120]. One hypothesis is that when senescence is delayed, grain protein is diluted by long-term carbohydrate accumulation that then causes the increased grain weight [121]. Another hypothesis indicates that protein synthesis cost more for plants compare to carbohydrate synthesis [122]. Research using stay green mutants have also found increase in biomass but not in seed yields. Seeds needed a longer seed filling period [115,123,124]. Different maize lines with different timing of leaf senescence showed higher nitrogen levels, even though the final yield was similar [125]. Early senescence in common wheat has also reported to be associated with higher minerals in grains [126]. Therefore, when breeding crops for earlier or later senescence timing, not only yield but also the nutritional facts and quality of yield should be taken under consideration; something that plants are naturally and wisely handling in nature by taking all the involving factors into account. This shows how choosing the right time for monocarpic senescence is very delicate as the final production of plants depends on it.

## 7. The Importance of Molecular Analyses of Senescence

The molecular mechanisms behind plant senescence are still mostly unknown because of their complexity. The complexity of senescence is evident in the fact that almost 10% of the total gene set from a genome is upregulated during senescence [15]. It has also been reported that more than 200 transcription factors in *A. thaliana* are involved in senescence, which shows a complex regulatory network being responsible for senescence [14,127,128]. However, over the past several years, using a variety of methods (e.g., microarray analyses and mutant screening), a large number of genes that are upregulated during and/or close to senescence have been identified in various plant species [129,130,131,132,133]. These genes are often called senescence association genes (SAGs). The leaf senescence database (LSD) (https://bigd.big.ac.cn/lsd/) is a comprehensive resource of senescence-associated genes (SAGs) and their corresponding mutants [128,134,135]. The current version (LSD 3.0) contains 5853 genes and 617 mutants from 68 species. LSD is a useful resource for study of senescence that also offers candidate genes for functional analyses. For example, using large number of screening mutants with altered senescence phenotype, researchers have performed functional analysis on candidate SAGs and reported upregulation of those genes during senescence and also delayed senescence associated with loss of function of those genes [136].

The expression patterns of candidate senescence genes have been reviewed and discussed in several articles [53,131,133,137,138,139], and it was not the intention in present review to reiterate those excellent references. Instead, the aim here was to focus mostly on the broader concept of senescence timing with an emphasis on the role of environmental clues that plants combine with internal factors in order to schedule senescence at proper time which is in favor of their fitness. However, a deeper understanding of scheduled senescence in plants and senescence synchrony will only be achieved when ecological data are supplemented with molecular analyses. The appropriate timing of senescence is essential for plant productivity, which involves expression of senescence associated genes (*SAG*s) at the proper time. Although many of these genes have been identified, our current understanding of the roles of most of them in regulating of senescence—especially whole-plant senescence—is still limited. Future approaches that utilize high-throughput molecular data and visualize dynamic transcriptome changes at senescence time in plants growing in different environmental conditions may facilitate a better understanding of regulatory genes encoding senescence timing and provide insights into its adjustment with ever changing environment.

## 8. “Premature Senescence”: A Forced, Unwanted Type of Senescence

Developmental senescence occurs even in stress-free conditions where plants are experiencing sufficient nutrition, optimal temperature, light, and moisture and are away from pathogen attacks. However, plants might experience another type of senescence called “premature senescence” which is triggered by high amounts of stress such as extremes in temperature, light and drought, soil salinity, mineral imbalance (especially nitrogen), pathogen attack, etc. that may cause insufficient growth in plants and further result in accumulation of nutrients in source due to reduced sink activity and therefore then lead to premature senescence [41,130,131,132,140,141]. For example, dark-induced senescence has experimentally been used for many times to induce uniform, rapid senescence [137].

However, even under the same amount of stress, plants do not respond to it the same, and endogenous factors such as age, reproductive development, and levels of regulators such as hormones may influence the capacity of the plant to enter senescence phase under stress. A review article [18] discussed the complex mechanisms of regulation of senescence in drought-stressed plants and proposed that senescence is triggered in response to environmental stress factors in some species, but not in others, and its onset also depends on the magnitude and severity of the stress and the growth phase in which the stress is imposed. They suggested that some species may have even evolved to withstand senescence under stress. Efficient senescence is essential to maximize viability in the next season or generation, but premature senescence, is a protective mechanism employed when plants are experiencing intense stress [15]. Some gene expression in drought has been reported [142,143], but the specific signaling pathways leading to senescence in stress is not clear yet.

## 9. Conclusions and Future Plans

The capacity of annual plants to control the pace of their life history events is one of their unique characteristics that enable them to thrive in almost every habitat on earth. Natural selection has led plants to evolve various mechanisms for regulating developmental plasticity and matching their life history phases with their environmental condition. With rapid pace of climate change, seasonal condition and therefore start and end of annual growing season will change for plants and it will influence the expression of their life history traits (such as germination timing, flowering, and senescence). This may cause strong natural selection on expression of those traits and also evolutionary responses that is dependent on genetic and epigenetic variation. Considering forecasted climate change, it is predicted that in order to avoid unfavored season and maximize fitness of the plants, selection will favor earlier germination and therefore earlier reproductive phase and senescence. How fast plants can adapt to this change to manage accomplishing their life stages successfully is an open question to be addressed.

Choosing the right senescence timing is especially very important for monocarpic plants as it affects their final production and fitness. It is important to understand how much control plants may have over their “time of death”, and it is promising that more researchers are now focusing on environmental impacts on senescence timing from life history perspective. The ability of time management in plants—especially timing senescence and final production—has great potential for improving agronomic traits, such as crop yields and post-harvest quality. In this review, we discussed how senescence timing is connected to all the previous life history stages and how they all have cascading effect on one another. Plants combine environmental signals with internal factors in order to make the best decision about their life schedules in favor of final outcome. Citing relative studies, we explained how whole-plant senescence is under season-dependent regulation more than age-dependent, and plants have the ability to synchronize with their seasonal environment. However, it remains to be determined whether this flexibility is heritable and what the possible genetic and epigenetic mechanism behind it is. Whether senescence timing is synchronously controlled in other plant species besides *A. thaliana* is also another novel question that should be addressed. A better understanding of regulatory mechanisms behind whole-plant senescence may help to take advantage of this trait for crop agriculture and manipulate them to accelerate yield time or even achieve synchronous harvest. Future studies may need to focus on both changes in phenological pattern based on plant’s environment and also the molecular mechanism behind it to define the direction and degree of plant adaptation and their flexibility to changing environment. Another question that arises is whether the molecular mechanisms of different type of senescence (organ senescence, reproductive senescence, and premature senescence) are similar. Hopefully, drawing attention to this direction will stimulate further research in this area.

## Data Availability

Not applicable.
